# Hippo pathway dysregulation in gastric cancer: from Helicobacter pylori infection to tumor promotion and progression

**DOI:** 10.1038/s41419-023-05568-8

**Published:** 2023-01-12

**Authors:** Beatrice Messina, Federica Lo Sardo, Stefano Scalera, Lorenzo Memeo, Cristina Colarossi, Marzia Mare, Giovanni Blandino, Gennaro Ciliberto, Marcello Maugeri-Saccà, Giulia Bon

**Affiliations:** 1 Clinical Trial Center, Biostatistics and Bioinformatics Unit, Department of Research, Diagnosis and Innovative Technologies, IRCCS Regina Elena National Cancer Institute, Rome, Italy; 2 Oncogenomic and Epigenetic Unit, Department of Research, Diagnosis and Innovative Technologies, IRCCS Regina Elena National Cancer Institute, Rome, Italy; 3 SAFU Laboratory, Department of Research, Advanced Diagnostic, and Technological Innovation, IRCCS Regina Elena National Cancer Institute, Rome, Italy; 4 Pathology Unit, Mediterranean Institute of Oncology, Viagrande, Italy; 5 Medical Oncology Unit, Mediterranean Institute of Oncology, Viagrande, Italy; 6 Department of Biomedical, Dental, Morphological and Functional Imaging Sciences, University of Messina, Messina, Italy; 7 Scientific Directorate, IRCCS Regina Elena National Cancer Institute, Rome, Italy; 8 Division of Medical Oncology 2, IRCCS Regina Elena National Cancer Institute, Rome, Italy; 9 Cellular Network and Molecular Therapeutic Target Unit, Department of Research, Diagnosis and Innovative Technologies, IRCCS Regina Elena National Cancer Institute, Rome, Italy

**Keywords:** Oncogenes, Tumour biomarkers, Cell growth

## Abstract

The Hippo pathway plays a critical role for balancing proliferation and differentiation, thus regulating tissue homeostasis. The pathway acts through a kinase cascade whose final effectors are the Yes-associated protein (YAP) and its paralog transcriptional co‑activator with PDZ‑binding motif (TAZ). In response to a variety of upstream signals, YAP and TAZ activate a transcriptional program that modulates cellular proliferation, tissue repair after injury, stem cell fate decision, and cytoskeletal reorganization. Hippo pathway signaling is often dysregulated in gastric cancer and in Helicobacter pylori-induced infection, suggesting a putative role of its deregulation since the early stages of the disease. In this review, we summarize the architecture and regulation of the Hippo pathway and discuss how its dysregulation fuels the onset and progression of gastric cancer. In this setting, we also focus on the crosstalk between Hippo and other established oncogenic signaling pathways. Lastly, we provide insights into the therapeutic approaches targeting aberrant YAP/TAZ activation and discuss the related clinical perspectives and challenges.

## Facts

- The Hippo pathway is a master regulator of tissue homeostasis acting through its final effectors YAP and TAZ.
- The Hippo pathway controls cytoskeletal remodeling, proliferation, tissue repair, and is involved in multiple cancer-related processes.
- The crosstalk between dysregulated Hippo pathway and established oncogenic avenues contributes to gastric cancer initiation, progression, and resistance to therapy.

## Open Questions

- Current evidence indicates Hippo dysregulation since Helicobacter pylori infection. Would effective targeting of this pathway at this stage prevent the onset of cancer?
- Although a number of drugs targeting the Hippo pathway have been developed, formal proof of clinical efficacy is still lacking. The dissection of the functional network connecting Hippo with other oncogenic pathways, and rationally designed trials aimed at assessing signaling modulation in response to a given drug, are required to develop novel therapeutic strategies targeting YAP/TAZ at multiple levels.

## Introduction

The Hippo signaling pathway is a key regulator of organ size and tissue homeostasis in animals. The first evidence connecting Hippo to organ size control, achieved by a coordinated regulation of proliferation and apoptosis, stemmed from *Drosophila* models. In this context, inactivating mutations or forced overexpression of key pathway genes resulted in the overgrowth of various organs and appendages (the “hippopotamus phenotype”) [1]. Later, studies in mice revealed that the effects of Hippo pathway manipulation are conserved in mammals [2, 3]. These sets of early evidence have also been instrumental in organizing the various components into a signal transduction pathway, characterizing the modality of their “vertical” and “lateral” interactions, and the way in which distal effectors operate to modulate the expression of target genes [4, 5].

The Hippo pathway is organized in two modules: (1) a core of serine-threonine kinases and adaptors with regulatory activity [6] and (2) a transcriptional effector module containing two related proteins, the transcriptional co-factor Yes-associated protein (YAP) and its paralog transcriptional co-activator with a PDZ-binding motif (TAZ) [7]. YAP/TAZ modulate pathway-responsive genes, prevalently through the interaction with TEA domain-containing sequence-specific transcription factors (TEAD1-4) [8–10].

Since the ablation of genes belonging to the regulatory module resulted in the onset of tumors, and a similar phenotype was observed with the overexpression of YAP, Hippo is considered a tumor-suppressor pathway, whose main function is the inhibition of the downstream YAP/TAZ proteins [11–13]. More than two decades of intense research revealed novel and unexpected functions and connections, such as those with the DNA damage response (DDR) machinery and the immune system [14–19]. Likewise, a wealth of studies conveyed the message that a variety of cues boosts tumor-promoting activities by dysregulating the Hippo signaling. As a result, the aberrant YAP/TAZ transcriptional activity was connected to invasion and distant dissemination [20–23], chemoresistance [24, 25], and maintenance/expansion of the cancer stem cell (CSCs) compartment [26–29].

Hippo pathway dysregulation is a common event in gastric cancer (GC), which is the sixth most diagnosed cancer worldwide and the third most common cause of cancer-related deaths [30]. First, it is involved in Helicobacter pylori (H. pylori)-induced processes that lead to gastric carcinogenesis [31, 32]. Second, downregulation of core regulatory kinases and upregulation/hyperactivation of YAP/TAZ effectors have been observed in GC [33–37]. Third, YAP/TAZ nuclear expression, denoting increased transcriptional activity, was associated with poor prognosis in GC in retrospective, hypothesis-generating studies [38, 39]. Pathway deregulation mainly occurs on a functional basis, given that mutations in core genes occur at a lesser extent than YAP/TAZ aberrant activation. Seminal large-scale genome studies carried out by The Cancer Genome Atlas (TCGA) network revealed that mutations and copy number variations in core genes occur in ~20–30% of gastric cancers (GC) (available at http://www.cbioportal.org). Nevertheless, the functional consequences of these alterations remain unclear.

In this review, we first introduce the organization and functional regulation of the Hippo signaling. Then, we provide an overview on the roles of Hippo pathway deregulation in GC, spanning from H. pylori-induced transformation to GC progression and resistance to anticancer treatments. Finally, current Hippo-targeting therapeutic strategies are discussed.

## Organization of the Hippo pathway

**Fig. 1:**
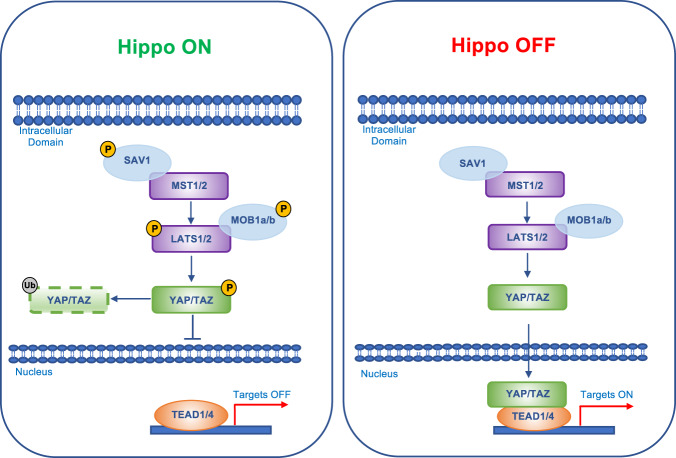
Hippo signaling pathway. Schematic diagram for the Hippo pathway core components and signaling. When Hippo signaling is ON, the activated Mammalian sterile 20-like kinase 1 and 2 (MST1/2) bind Salvador homolog 1 (SAV1) and phosphorylate/activate Large tumor suppressor 1 and 2 (LATS1/2) and their cofactors MOB kinase activator 1A and 1B (MOB1A/B). The activated LATS/MOB phosphorylate YAP/TAZ which results in its cytoplasmic retention by 14-3-3 protein and proteasomal degradation. As a result, YAP/TAZ cannot interact with TEAD in an active transcriptional unit (targets OFF). When Hippo signaling is OFF, MST1/2 and LATS kinases are inactive. Unphosphorylated YAP/TAZ translocate into the nucleus and interact with TEAD (TEA Domain transcription factor) to induce the transcription of target genes (targets ON).

In mammals, the central axis of the Hippo pathway comprises a phosphorylation cascade in which the serine/threonine kinases Mammalian sterile 20-like kinase 1 and 2 (MST1/2) bind their cofactor Salvador homolog 1 (SAV1) and phosphorylate and activate Large tumor suppressor 1 and 2 (LATS1/2). LATS1/2 and their cofactors MOB kinase activator 1A and 1B (MOB1A/B) phosphorylate and inactivate YAP/TAZ, promoting their cytoplasmic retention and proteasomal degradation [5]. Conversely, when YAP and TAZ are in the “ON” form, they translocate into the nucleus and bind TEAD1–4 transcription factors to induce the expression of target genes [40] (Fig. 1).

Multiple serine residues have been involved in YAP/TAZ phosphorylation downstream the Hippo pathway (YAP: S61, S109, S127, S164, and S381; TAZ: S66, S89, S117, and S311) [4]. For instance, LATS1/2-induced phosphorylation of YAP and TAZ on ser-127 and ser-89 residues, respectively, results in their binding to 14-3-3 proteins and cytoplasm retention [8]. LATS1/2 also phosphorylates YAP and TAZ on ser-381 and ser-311, respectively, determining their polyubiquitination and proteasome degradation. Furthermore, TAZ ser-311 phosphorylation induces phosphorylation on ser-314 by Casein kinase 1 (CK1), promoting TAZ interaction with the SCF multisubunit complex (Skp1, Cullins, F-box proteins) E3 ubiquitin ligases, and thus its degradation [41].

Under basal conditions, the Hippo pathway is regulated by a variety of stimuli acting at different levels of the signaling cascade that ensures tissue homeostasis. These regulatory mechanisms are mainly mediated by mechanical cues (e.g., cell–cell interactions, cell density, and cell polarity), cell surface receptors and adhesion molecules. A further level of regulation acts downstream, and inhibits YAP-TEAD interaction. These include Vestigial-like 1-4 (VGLL1-4) [42] and Runt-related transcription factor 3 (RUNX3) [43], which bind TEAD in a competitive dynamic.

### Regulation by mechanical cues

Among proteins involved in mechanical cues, the tight junction protein Zona occludens-2 (ZO-2) mediates the regulation of Hippo activity by signals released from cell-cell interactions. ZO-2 represents the main regulator of YAP/TAZ localization upon loss of cell-cell contact. In low cell density in vitro, ZO-2 promotes YAP nuclear localization, whereas YAP is enriched at the plasma membrane in confluent cells [44]. Also, Neurofibromin-2 (NF2) acts as a regulator of Hippo kinases implicated in cell-cell contact inhibition. NF2 is localized at tight and adherent junctions, and when high cell density is reached, it induces the activation of Hippo core kinases by two mechanisms: (1) interaction with LATS and stimulation of its complexing with SAV, and (2) promotion of LATS-YAP interaction by facilitating the assembly of scaffold proteins [45]. Moreover, NF2 regulates TEAD activity by affecting its palmitoylation status [46]. Overall, these regulations result in YAP/TAZ inhibition to limit cell proliferation.

The adaptor protein Scribble is localized at the plasma membrane, where it plays a key role in the regulation of cell polarity. Scribble promotes the formation of a complex including MSTs, LATS, and TAZ, thereby enhancing the Hippo cascade activity [26].

The FAT family of atypical cadherins control a form of tissue organization known as planar cell polarity via the Hippo pathway [47]. Mechanistically, FAT1 complexes with and promotes the assembly of the core Hippo signaling complex, leading to YAP1 phosphorylation and inactivation. Interestingly, FAT1 loss-of-function mutations are frequent in cancer and result in YAP1 activation [48].

Variations in extracellular matrix (ECM) stiffness or cell stretching regulate Hippo signaling by YAP/TAZ phosphorylation. This occurs via the apical crumbs complex (CRB), the angiomotin family (AMOTs) components, and proteins linking cadherins to the actin cytoskeleton, such as α-catenin [49–52]. AMOTs sequester YAP through direct binding, leading to YAP retention to the plasma membrane. F-actin polymerization competes for AMOT130 binding, resulting in the release of YAP and its nuclear accumulation [51].

### Regulation by cell surface receptors

Cell surface receptors implicated in the regulation of Hippo pathway include G-protein coupled receptors (GPCRs) [53] and the Leukemia inhibitory factor receptor (LIFR) [54]. GPCR is the largest family of surface receptors comprising more than 800 members, and are involved in a wide variety of physiological processes by responding to a plethora of endogenous ligands (e.g., hormones, neurotrasmitters, chemokines). In the context of Hippo regulation, stimulation of G12/13-coupled receptors by lysophosphatific acid (LPA) and sphingosine 1-phosphophate (S1P) inhibit LATS1/2, thereby activating YAP/TAZ. In contrast, metabolic hormones such as glucagon or epinephrine activate LATS1/2 through GPCRs, hence inhibiting YAP/TAZ function. Thus, depending on the engaged G protein, signaling from GPCR can either activate or inhibit the Hippo pathway, linking the pathway to many upstream regulatory signals acting at the systemic level.

LIFR is a multifunctional cytokine involved in cancer promotion, and an activator of the Hippo pathway [54]. LIFR has been proposed as a prognostic marker in breast cancer, where its loss is associated with metastasis and poor clinical outcomes. In studies exploiting cellular and mouse models, LIFR ectopic expression, or treatment with its ligand leukemia inhibitory factor (LIF), activates the Hippo cascade leading to phosphorylation and cytoplasmic retention of YAP, thereby suppressing metastasis.

### Regulation by crosstalk with other signaling pathways

**Fig. 2:**
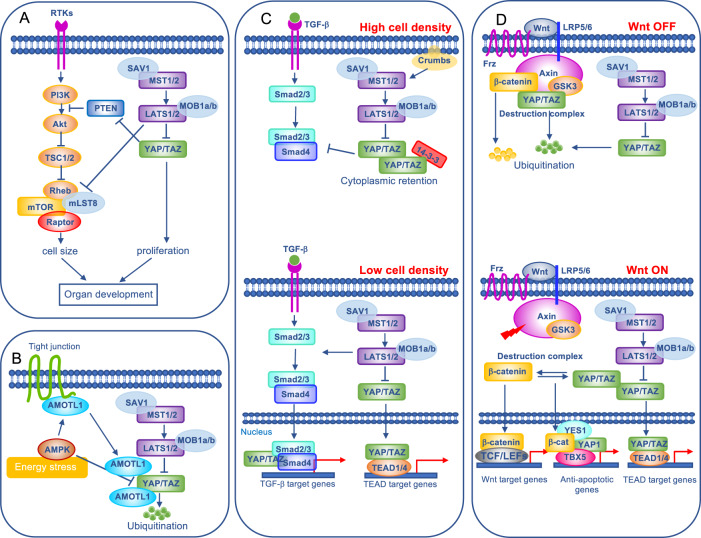
Crosstalk of the Hippo pathway with other signaling networks. **A** The functional connection between mTOR (regulating cell size) and Hippo (regulating proliferation) pathways is essential for the proper organ development. At the basis of this crosstalk, YAP can downregulate PTEN (Phosphatase and tensin homolog), whereas LATS1/2 (Large tumor suppressor kinase 1/2) can phosphorylate Raptor, resulting in inhibition of mTOR signaling. **B** Under energy stress condition, the AMP-activated protein kinase (AMPK) phosphorylates and stabilizes AMOTL1 (Angiomotin Like 1), together with inducing both direct and LATS-mediated YAP phosphorylation, leading to YAP inhibition. **C** In conditions of high cell density, the Hippo pathway is activated and cytoplasmic YAP/TAZ retain TGFβ (transforming growth factor β)-activated SMAD family members 2/3-4 (SMAD 2/3-4) in the cytoplasm. At low cell density, YAP/TAZ and SMAD 2/3-4 translocate to the nucleus to cooperatively induce transcription. **D** YAP and TAZ are members of β-catenin destruction complex, responsible for β-catenin inactivation. Upon activation of the Wnt pathway, YAP/TAZ and β-catenin enter the nucleus and synergistically induce Wnt and TEAD (TEA Domain transcription factor) target genes. Moreover, by complexing with TBX5 (T-box transcription factor 5), they induce anti-apoptotic genes.

Interactions of Hippo with other signaling pathways is extensively investigated (Fig. 2), and highlights the complex molecular machinery that intersects the pathway. Under homeostatic conditions, positive and negative signals are balanced to ensure proper signaling activity [55].

The crosstalk with Mammalian target of rapamycin (mTOR) pathway is particularly relevant. Indeed, the balanced activity of Hippo pathway and mTOR signaling ensures cellular homeostasis and dictates proper organ development. For instance, YAP downregulates the tumor suppressor Phosphatase and tensing homolog (PTEN), a negative regulator of mTOR, resulting in increased cell size [56]. Later, LATS1/2 have been demonstrated to play a key role in coordinating the two pathways by phosphorylating Raptor, a component of mTORC1, impairing its interaction with Rheb, and then attenuating mTORC1 activation [57].

YAP and TAZ are binding partners of SMAD family members, the main signal transducers of the transforming growth factor beta (TGF-β) and bone morphogenic protein (BMP) growth factors families [58]. Under high cell density conditions, CRB elements induce Hippo pathway activation. In this circumstance, cytoplasmic YAP and TAZ abrogate TGFβ-dependent activation of SMAD 2/3-4 by cytoplasmic sequestration of the latter. By contrast, at low cell density YAP and TAZ are active and mainly localized in the nuclear compartment, enabling TGFβ to phosphorylate SMAD 2/3-4 and consequently their nuclear translocation [49].

Next, an elegant model was proposed to explain the signaling framework involving Hippo and Wnt, which is central in organ size control and tumor suppression [59]. By using cell and mouse models, the authors have demonstrated that YAP and TAZ are integral components of the β-catenin destruction complex. Upon activation of Wnt signaling, both YAP and β-catenin are dislodged from the destruction complex, and accumulate in the nucleus, where they synergistically regulate proliferation and differentiation. In this scenario, the release of YAP/TAZ from the complex is a critical event that enhances Wnt/β-catenin signaling. In support of the key role played by YAP/β-catenin crosstalk in cancer, YAP and the transcription factor TBX5 form a complex with β-catenin that induces the transcription of anti-apoptotic genes. YAP phosphorylation by the Src family kinase c-Yes (YES1) is required for this regulation [6].

In addition, crosslinking with metabolic pathways suggests a functional role for the Hippo pathway in cellular metabolism. These include glucose, fatty acids, the mevalonate pathway, hormones acting through GPCRs, and energy sensor pathways. For instance, AMP-activated protein kinase (AMPK) regulates the Hippo pathway by three different mechanisms, leading to YAP suppression: (i) through phosphorylation and stabilization of Angiomotin Like 1 (AMOTL1) [60]; (ii) via phosphorylation of YAP at Ser61, Ser94, and Thr119, together with enhancing LATS-induced phosphorylation of YAP at Ser127 [61]; and (iii) via competitive interaction with YAP that disrupts YAP-TEAD complex [62].

Glucose metabolism triggers YAP-dependent transcription through different mechanisms related to the glycolysis pathway. Phosphofructokinase 1 (PFK1), a key rate-limiting enzyme of glycolysis, interacts with TEADs and potentially regulates Hippo functional output [63]. In the presence of high levels of glucose, YAP *O*-GlcNAcetylation by *O*-GlcNAc transferase (OGT) prevents LATS-induced YAP phosphorylation, allowing *O*-GlcNAcetylated YAP to induce transcriptional activation in the nucleus [64]. Similarly, GlcNAcetylation of AMOTs, induced by high glucose levels, contributes to YAP nuclear accumulation and activation [65].

The mevalonate pathway, responsible for the generation of isoprenoids, is a further regulator of the Hippo pathway. The geranylgeranyl pyrophosphate produced during the mevalonate cascade activates Rho GTPases that, in turn, trigger YAP/TAZ nuclear localization by inhibiting their phosphorylation [66]. Collectively, evidence indicates that multiple pathway-extrinsic forces converge on the Hippo cascade, spanning from proliferative signals to metabolic cues.

### Hippo signaling and cell death pathways

As part of the complex network of developmental and environmental signals that control tissue homeostasis in *Drosophila*, activation of the Hippo pathway is required for cell death response elicited by ionizing radiation and p53 ectopic expression [14]. In mammals, the multifunctional nature of the Hippo pathway is denoted by the very different outcomes that can be reached through YAP/TAZ activation: proliferation/oncogenic transformation [11, 12, 20–23, 28] and cell death/tumor suppression [67–71]. In tumors, aberrant YAP activation is associated with multiple cancer-related processes, and the YAP/TEAD transcriptional unit stimulates the expression of pro-survival and anti-apoptotic genes. However, under DNA damage stress conditions, nuclear YAP interacts with the p73 transcription factor enhancing the transcription of pro-apoptotic genes, such as *p53AIP1* [67], *Bax* [68], *DR5* [69], and *PUMA* [70]. A multitude of Hippo regulators have been reported to be involved in activating YAP by either impeding and/or inducing apoptosis. Among the latter, the tumor suppressor Ras association domain family 1 isoform A (RASSF1A) enhances MST2-LATS1 interaction leading to YAP nuclear translocation and interaction with p73 [70]. Similarly, the transcription factor Early growth response-1 (EGR-1) interacts with YAP to induce clonogenic cell death in prostate carcinoma cells [72].

YAP/TAZ activators that impede apoptosis include the transcription factor AP-2 Gamma (TFAP2C), WW domain binding protein 5 (WBP5), nuclear factor kappa B kinase subunit epsilon (IKBKE), cAMP response element-binding (CREB) protein, and Forkhead box protein A1 (FOXA1). The mechanisms through which these regulators affect apoptosis in a YAP-dependent manner are described in detail elsewhere [73].

Overall, the conflicting nature of the findings that link Hippo signaling to cell death pathways suggests that cellular context, and the specific network of pathway regulations, might be key determinants of the final outcome.

## The role of Hippo pathway dysregulation in gastric cancer

### Murine models of gastric carcinogenesis

In cancer, aberrant YAP/TAZ activation can result from mechanotransduction dysregulations, as a consequence of alterations in cell polarity/ECM stiffness, and/or loss of cell-cell contact inhibition [74, 75]. Similarly, YAP/TAZ control tumor progression and distant dissemination through multiple mechanisms, including autoregulatory feedback loops and pathway crosstalk [76–81], as well as co-repressor functions towards tumor-suppressor genes [82–84].

The oncogenic role of YAP/TAZ has exhaustively been elucidated by studies in engineered mouse models of liver tumorigenesis [3, 45, 85–89]. Liver-specific deletion of MST1/2 and SAV1 was sufficient to trigger liver tumorigenesis, and transcriptional profiling revealed the enrichment of Hippo-regulated genes involved in immune and inflammatory responses [87, 89]. Similarly, conditional expression of YAP in the mouse liver induced massive hepatomegaly followed by the onset of hepatocellular carcinoma. A microarray-based analysis at the stage of hyperplasia revealed that YAP induced the transcription of pro-proliferative genes such as *ki-67*, *C-Myc*, *SOX4*, *H19*, and *AFP*, coupled with anti-apoptotic genes such as *BIRC5/survivin*, *BIRC2/cIAP1*, and the BCL2 family gene *MCL1* [3]. While established models of liver tumorigenesis demonstrated the involvement of Hippo in liver/biliary tumors, a direct nexus with GC was recently described. Indeed, YAP/TAZ activation, triggered by conditional knockout of LATS1/2 in LGR5-expressing pyloric stem cells, was shown to initiate gastric tumorigenesis in mice. MYC was identified by RNA-sequencing analyses as a key downstream mediator of this process, and a direct transcriptional target of YAP [90]. Therapeutically, blocking the YAP-TEAD interaction by the use of a peptide mimicking the role of VGLL4, suppressed tumor growth in xenograft and carcinogen-induced murine GC models [91]. Collectively, these data suggest that Hippo dysregulation is associated with the onset of GC via stem cell expansion and oncogenic cooperation mechanisms.

### Hippo pathway and Helicobacter pylori-mediated gastric carcinogenesis

**Fig. 3:**
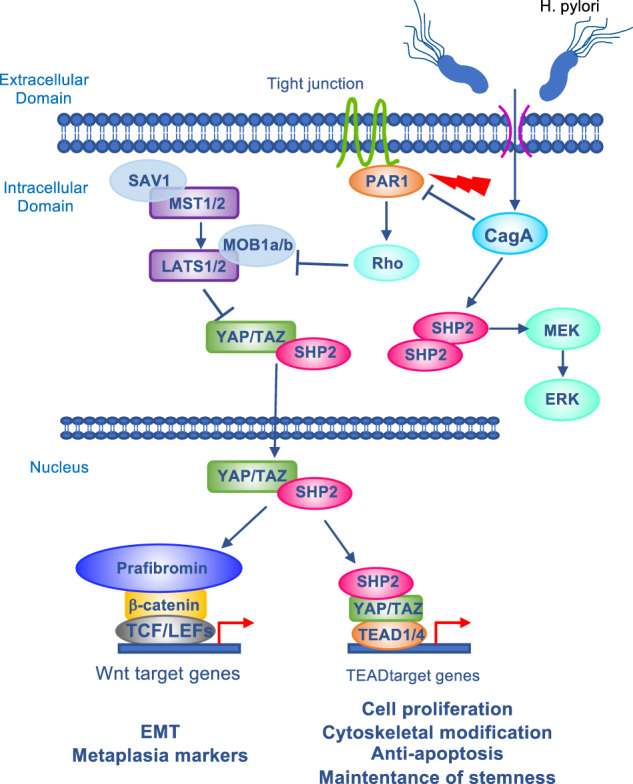
Mechanisms of YAP/TAZ activation induced by Helicobacter pylori infection. The H. pylori-secreted oncoprotein Cytotoxin-associated gene A (CagA) activates YAP/TAZ through different mechanisms. CagA mediates the crosslink between Hippo and Wnt pathways by inducing SHP2 (SH2 containing protein tyrosine phosphatase-2), that activates RAS-ERK signaling and promotes YAP/TAZ nuclear translocation and activation of TEAD-regulated genes. Once in the nucleus, SHP2 interacts with parafibromin/β-catenin in a transcriptionally active complex, which induces Wnt target genes. Moreover, by inhibiting Partitioning-defective 1 (PAR1), CagA induces the disruption of tight junctions, resulting in YAP/TAZ activation.

H. pylori infection is the strongest known risk factor for stomach cancer. Through various virulence factors, H. pylori leads to chronic inflammation by activating a number of pathways, both in gastric epithelial cells and in immune cells recruited to the site of infection. The resulting onset of chronic gastritis is a major step in the initiation and development of gastric cancer. As part of this process, dysregulation of cell-cell junctions and increased stiffness of the gastric wall contribute to the disruption of Hippo signaling and downstream malignant transformation [92–94]. The major effector is the H. pylori-secreted oncoprotein Cytotoxin-associated gene A (CagA) [95]. In gastric cells, CagA binds to the tyrosine phosphatase SH2 containing protein tyrosine phosphatase-2 (SHP2), stimulating its activity. In the cytoplasm, SHP2 is required for the full activation of the RAS-ERK signaling. Non-phosphorylated YAP and TAZ have been reported to physically interact with SHP2 promoting its nuclear translocation [96], which stimulates TEAD-regulated genes. Nuclear SHP2 also induces the formation of the transcriptionally active parafibromin/β-catenin complex, which in turn induces Wnt target genes, thereby promoting the crosstalk between Hippo and Wnt/catenin pathways. In addition, CagA induces the disruption of tight junctions through the inhibition of Partitioning-defective 1 (PAR1), a serine/threonine kinase involved in the regulation of cell polarity [97]. Thus, CagA induces loss of cell-cell contact, with consequent increase in YAP/TAZ-mediated gene transcription (Fig. 3). Accordingly, the transcriptomic analyses of gastric epithelial cells after H. pylori infection revealed YAP upregulation and activation of the YAP/TEAD transcriptional machinery, as indicated by the increase of target pro-survival and pro-proliferation genes [31]. Under the same conditions, LATS2 is overexpressed in an attempt of the host cells to tolerate the H. pylori-induced alterations in the gastric epithelium. The involvement of CagA in these processes is demonstrated by the lack of YAP1/LATS2 deregulations following infection of gastric cells with CagA-mutant strains of H. pylori. Functionally, the activation of YAP/TEAD in H. pylori-infected gastric epithelial cells is sufficient to promote epithelial to mesenchymal transition (EMT), and the acquisition of metaplasia markers [31, 98].

Likewise, TAZ nuclear expression and TAZ/TEAD activity are required for H. pylori-induced EMT and CSC-related tumorigenic properties in gastric epithelial cell lines [32]. TAZ and zinc-finger E-box binding homeobox 1 (ZEB1), a transcription factor closely associated with EMT, were co-overexpressed in cells with a mesenchymal phenotype in vitro, in areas of hyperplasia in H. pylori-infected patients, as well as at the invasive front of gastric carcinoma. Moreover, the depletion of TAZ reduced ZEB1 expression, mitigated the EMT phenotype, and inhibited H. pylori-induced invasion and tumorsphere formation.

Overall, these studies indicate that the Hippo pathway is involved in H. pylori-induced pro-tumorigenic properties, and provide the rationale for exploring its therapeutic targeting as a tumor-preventing strategy.

### Hippo pathway in gastric carcinogenesis

Beyond H. pylori, several studies documented the link between Hippo and GC. Reduced expression of MST1/2 and LATS1/2 kinases and elevated expression of YAP/TAZ effectors are frequently observed in GC [33–37]. Low LATS1 expression is associated with lymph node metastasis, poor prognosis, and disease recurrence in GC patients. The ectopic expression of LATS1 decreased proliferation and invasion of GC cells in vitro and impaired tumor growth and metastatization in vivo through YAP inhibition, whereas LATS1 depletion rescued the invasive phenotype [34]. Also, Tang Y and colleagues have reported a mechanism responsible for MST1/2 inactivation. According to this model, Striatin 3 (STRN3), an essential regulatory subunit of Protein phosphatase 2A (PP2A), promotes MST1/2 recruitment and dephosphorylation, resulting in YAP activation. Consistently, STRN3 is highly expressed in GC patients, where it is associated with YAP activation and poor prognosis [99].

YAP is overexpressed in high-grade dysplasia, gastric adenocarcinoma, and metastatic disease [35, 36]. Importantly, overexpressed YAP is mainly localized at the cytoplasm in the early stages of GC, whereas elevated levels of nuclear YAP are observed at advanced tumor stages. In line with this, nuclear accumulation of YAP is associated with poor survival particularly in early stage GC patients [38]. In GC cell lines, YAP depletion results in decreased proliferation and invasion/migration. By contrast, YAP ectopic expression promotes proliferation, anchorage-independent growth, and invasive properties by activation of the mitogen-activated protein kinase (MAPK) signaling pathway [38, 100]. Similarly, the overexpression of TAZ is associated with EMT and reduced survival in GC patients [101]. Interestingly, TAZ expression characterizes gastric signet ring cells carcinoma, a distinct type of poorly differentiated GC associated with earlier onset and worse prognosis [102].

YAP/TEAD-negative regulators are often deregulated in GC. The mRNA levels of VGLL4 are downregulated in a significant fraction of GC cases and are inversely correlated with tumor stage and lymph node metastasis [91]. The loss of RUNX3 tumor-suppressor is observed in 60% of GC specimens, and is associated with higher TEAD-YAP expression both in GC patients and cell lines [43]. Likewise, RUNX2 plays a key role in promoting GC invasion and metastatization through YAP activation [103].

Adding a further level of complexity, YAP-activating mechanisms involved in GC also include upregulation of Methyltransferase3 (METTL3) [104], AMOTL1 [105], Fibroblast growth factor receptor type 2 (FGFR2) [106], Nucleolar and spindle associated protein 1 (NUSAP1) [107], Microtubule-associated monooxygenase, calponin and LIM domain containing 2 (MICAL2) [108], and Interferon regulatory factor 3 *(*IRF3) [109], as well as loss of PTEN [110] and MST4 kinase [111]. Overall, the variety of mechanisms that account for aberrant YAP/TAZ activation in GC unveil its critical contribution to GC tumorigenesis (summarized in Table 1).

**Table 1:** Hippo pathway deregulation events reported in gastric cancer.

Type of deregulation	Reference	Functional relevance	Proposed mechanism
LATS1 downregulation	Zhang J, *Oncotarget 2016* [34]	Lymph node metastasis, poor prognosis, recurrence in GC patients	
MST1/2 inactivation	Tang Y, *Cancer Cell 2020* [99]	STRN3 high expression is associated with poor prognosis in GC patients	STRN3 promotes MST1/2 dephosphorylation, resulting in YAP activation
YAP/TAZ overexpression/activation	Hu X, *Pathol Oncol Res 2014* [35] Da CL, *World J gastroenterol 2009* [36]	Progression, metastasis, poor prognosis in GC patients	
	Kang W, *Clin Cancer Res 2011* [38]	Nuclear YAP expression is associated with poor survival, especially in GC patients with early-stage disease	YAP induces the activation of the MAPK signaling pathway
	Liu H, *Oncol Lett 2019* [100]	YAP depletion reduces the viability, migration, and invasion of GC cell lines	YAP inhibits the endoplasmic reticulum stress pathway in an ERK-dependent manner
	Giraud J, *Int J Cancer 2020* [101]	TAZ expression is associated with poor overall survival in nonmetastatic GC patients	
	Zhou W, *J Oncol 2021* [104]	METTL3 sustains proliferative, migrative, and invasive properties of GC cell lines	METTL3 induces the mRNA levels of YAP as well as YAP target genes
	Zhou Y, *Oncogene 2020* [105]	High AMOTL1 expression is associated with advanced stage and poor overall survival in GC patients. AMOTL1 depletion impairs invasion and MAPK-dependent proliferation in GC cell lines	AMOTL1 interacts with YAP promoting its nuclear translocation
	Zhang J, *Oncogene 2020* [106]	High FGFR2 expression is associated with advanced stage and predicts poor survival in GC patients. FGFR2 depletion inhibits the growth and cell cycle progression in GC cell lines	FGFR2 induces YAP activation through MAPK-c-Jun signaling
	Guo H, *Front Oncol 2021* [107]	NUSAP1 upregulation is associated with unfavorable clinical outcomes in GC patients. NUSAP1 depletion impairs oncogenic properties of GC cell and mouse models	NUSAP1 acts as a positive regulator of YAP protein stability
	Qi C, *Oxid Med Cell Longev 2021* [108]	High MICAL2 expression is associated with poor overall survival in GC patients. The knockdown of MICAL2 attenuates proliferation in GC cell lines	MICAL2 promotes YAP nuclear translocation through ROS generation
	Jiao S, *J Exp Med 2018* [109]	IRF3 is often upregulated in GC patients. High levels of both IRF3 and YAP predict lower survival in GC patients	IRF3 interacts with both YAP and TEAD4 in the nucleus to enhance their occupancy on target genes
	Xu W, *J Exp Clin Cancer Res 2018* [110]	PTEN inactivation promotes the proliferationa and migration of GC in vitro and in vivo	PTEN inactivation induces YAP-TEADs activity by abolishing MOB1-LATS1/2 interaction and links Hippo and PI3K/Akt patways to promote GC
	An L, *J Exp Med 2020* [111]	Loss of MST4 is associated with poor prognosis in GC patients and promotes gastric tumorigenesis in mouse model	MST4 phosphorylates YAP at Thr83 leading to its cytoplasmic retention and inactivation
YAP/TEAD-repressors downregulation	Qiao Y, *Oncogene 2016* [43]	RUNX3 downregulation is associated with high TEAD-YAP expression, that predicts lower survival in GC patients	RUNX3 inhibits YAP-TEADs activity by binding TEAD and impairing its DNA-binding ability
	Jiao S, *Cancer Cell 2014* [91]	VGLL4 downregulation is inversely correlated with tumor size and stage and lymph node metastasis	VGLL4 competes with YAP for TEADs binding, resulting in inhibition of YAP-TEADs transcriptional activity

*AMOTL1* angiomotin like 1, *c-Jun* JUN proto-oncogene, *ERK* extracellular-signal regulated kinase, *FGFR2* fibroblast growth factor receptor 2, *GC* gastric cancer, *IRF3* *Interferon regulatory factor 3,* *Akt* AKT serine/threonine kinase 1, *LATS1/2* large tumor suppressor kinase 1/2, *MAPK* mitogen-activated protein kinase, *METTL3* methyltransferase 3, N6-adenosine-methyltransferase complex catalytic subunit, *MICAL2* microtubule associated monooxygenase, calponin and LIM domain containing 2, *MOB1* MOB kinase activator 1, *MST1/2* mammalian STE20-like protein kinase 1/2, *MST4* mammalian STE20-like protein kinase 4, *NUSAP1* nucleolar and spindle associated protein 1, *PI3K* phosphoinositide 3-kinase, *PTEN* phosphatase and tensin homolog, *ROS* reactive oxygen species, *RUNX3* RUNX family transcription factor 3, *STRN3* Striatin 3, *TEAD* TEA domain transcription factor, *VGLL4* vestigial like family member 4, *YAP1* yes-associated protein 1.

**Fig. 4:**
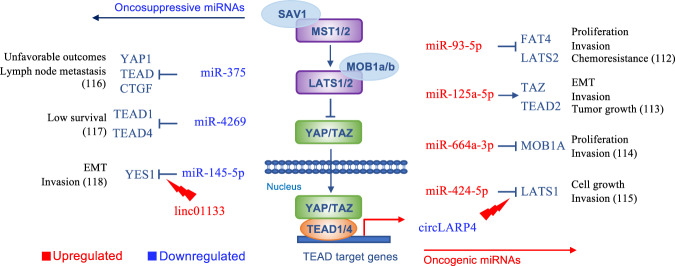
miRNAs implicated in Hippo pathway dysregulation in gastric cancer. Oncogenic miRNAs activating YAP/TAZ signaling are often upregulated (in red) in human GC. By contrast, YAP/TAZ inhibiting oncosuppressive miRNAs are reported to be downregulated (in blue) in GC. For each miRNA are indicated the Hippo target/targets, and the functional effects of its/their regulation. The stable circular RNA LARP4 (circLARP4) can reverse the oncogenic role of miR-424-5p, resuming YAP phosphorylation and retention in the cytoplasm, and is often downregulated in GC. Similarly, the long intergenic non-coding RNA linc01133 competes with miR-145-5p to promote YES1-dependent YAP1 nuclear translocation and is upregulated in GC tissues.

Next, many microRNAs (miRs) are involved in deregulations of the Hippo pathway in GC, including both oncosuppressive and oncogenic miRs. Among the latter miR-93-5p [112], miR-125a-5p [113], miR-664a-3p [114], and miR-424-5p [115] deserve to be mentioned. These miRs mainly act through inhibition of Hippo core kinases, except miR-125a-5p, that induces TAZ/TEAD2 activation. Likewise, oncosuppressive miRNAs are often downregulated in GC, and associated with inferior survival outcomes. These include miR-375 [116], miR-4269 [117], and miR -145-5p [118], which share inhibitory effects on YAP/TEAD transcriptional activity. The targets and functional effects of miRNAs-dependent regulations of Hippo signaling are reported in Fig. 4.

### Hippo pathway, stemness and therapeutic resistance in gastric cancer

Among the tumor-promoting functions of the Hippo pathway is the maintenance of the CSC compartment. YAP activation and nuclear localization are required for Stearoyl-CoA desaturase-1 (SCD1)-dependent induction of gastric CSCs (GCSCs) [119]. SCD1 is a key enzyme in fatty acid metabolism, that is involved in tumor progression and metastatization across a range of solid tumors [120]. SCD1 upregulation in metastatic GC was correlated with the expression of YAP and TEAD1, and YAP nuclear expression was reduced in tumor samples characterized by low levels of SCD1. Moreover, the depletion of YAP reduced the self-renewal and invasive capabilities induced by SCD1 in GCSC models in vitro. Accordingly, transcriptomic analyses revealed the enrichment of a Hippo-related gene signature in CD44 + gastric CSCs, with a plethora of target genes overexpressed (*AREG*, *BIRC5*, *CCND1*, *CCX2*, *CYR61*, *ID1*, *IGFBP3*, *JAG1*, *LATS2*, *MYC*, and *SMAD7*). Interestingly, TEAD1 and TEAD4 were also found upregulated in CD44 + cells compared to CD44- cells (non-CSCs), while VGLL4 and RUNX3 were downregulated [101].

The connection between deregulated Hippo signaling and stem-like properties functionally links YAP/TAZ aberrant activity to therapy resistance. Indeed, the CSC-like subpopulation exhibits self-renewal and differentiation capabilities that contribute to resistance. A noteworthy example is the higher activity of YAP/TAZ/TEAD detected in CD44 + gastric CSCs cells resistant to conventional chemotherapy [101]. In this biological context, the GPCR Protease-activated receptor-1 (PAR1) induces stemness and multi-drug chemoresistance by promoting Rho-dependent inactivation of LATS1/2, which results in YAP activation [51, 121]. Recently, the receptor tyrosine kinase Erythropoietin-producing hepatocellular receptor A2 (EphA2) has been implicated in chemotherapy resistance through YAP activation [122]. Importantly, higher co-expression of EphA2 and nuclear YAP in GC tumors was correlated with tumor relapse. A further mechanism linking YAP activation to chemotherapy resistance involves Annexin A6. Once released in extracellular vesicles (EV) from cancer-associated fibroblasts (CAFs) in the extracellular matrix, Annexin A6 activates a β1 integrin-focal adhesion kinase (FAK)-YAP axis. In a peritoneal metastasis mouse model, CAF-EV induced resistance to cisplatin, in a process that was attenuated by YAP inhibition [123].

Also, the oncogenic crosstalk between Hippo and Wnt was connected to chemoresistance and unfavorable survival outcomes in GC. In a study including 86 patients with advanced GC treated with first-line chemotherapy, a significant association between nuclear TAZ expression and Wnt mutations was revealed [124]. Patients harboring both nuclear TAZ and Wnt mutations had an increased risk of disease progression and death. Interestingly, concomitant YAP expression and *TP53* mutations are associated with better survival outcomes in patients receiving first-line chemotherapy [125]. A possible explanation for this apparent paradox is that YAP/p53 cooperatively induce a pro-proliferative program that may render cancer cells more vulnerable to cytotoxic therapies.

The molecular mechanisms by which YAP determines resistance to cancer therapies are being thoroughly investigated. By employing GC cell models, it has been reported that YAP impairs cisplatin efficacy by inducing Epidermal growth factor receptor (EGFR) expression and its downstream signaling [126]. A recent study described the involvement of YAP in the mechanisms by which lymph node metastasis (LNM)-derived GC (LNM-GCs) cells reprogrammed bone-marrow-derived mesenchymal stem cells (BM-MSCs) towards tumor-promoting phenotype and function, via secreted exosomes. Specifically, exosomal Wnt5a induced YAP activation by dephosphorylation in BM-MSCs [127]. These cancer-associated MSCs are fundamental components of the tumor microenvironment (TME), that dictates cancer cells resistance to therapies by exerting immuno-suppressive functions and activating resistance-related mechanisms in cancer cells [128]. In line with this, in a transwell co-culture system, BM-MSCs increased cisplatin resistance in CD133 + gastric CSCs through activation of the PI3K/AKT pathway [129].

Regarding targeted therapies, the activation of a HER4-YAP1 axis-induced EMT, and has been proposed as a mechanism accounting for trastuzumab resistance. Indeed, HER4, its phosphorylated form, and the mesenchymal marker vimentin were found to be upregulated in trastuzumab-resistant cell and mouse models, whereas epithelial markers displayed an opposite pattern [130]. Interestingly, YAP depletion rescues the expression of epithelial markers while lowering that of mesenchymal proteins in trastuzumab-resistant cells, indicating that YAP is a downstream effector of HER4 and is required for the regulation of EMT.

## Targeting strategies

**Fig. 5:**
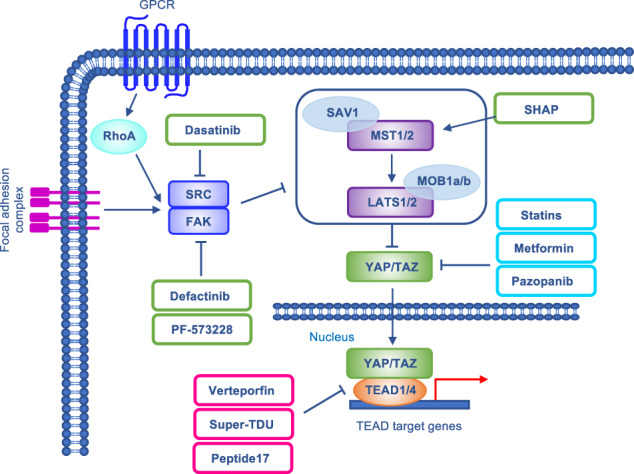
Drugs targeting the Hippo pathway. Hippo-targeted drugs act at different levels of the signaling cascade: some of them directly target the YAP-TEAD complex (red squares), while others inhibit YAP/TAZ nuclear localization (sky blue squares) or act at the level of upstream regulators, mainly linked to mechanical cues (green squares).

YAP/TAZ are attractive drug targets for cancer therapy in consideration of their high expression in many cancer cell types and their involvement in cancer progression and resistance. Given the limited access of macromolecules (e.g., antibodies) to the nucleus, where YAP/TAZ act as transcriptional cofactors, the search for targeted inhibitors is mostly focused on small molecules. The proposed therapeutic strategies encompass the disruption of YAP/TAZ-TEAD interaction, inhibition of YAP/TAZ nuclear localization, and inhibition of mechanical cues (Fig. 5). An updated list of clinical trials on Hippo inhibitors in gastrointestinal cancers has recently been reported elsewhere [131].

### Drugs targeting YAP-TEAD complex

In 2012, Liu-Chittenden and colleagues screened more than 3,300 FDA-approved drugs for their ability to disrupt the physical association between YAP and TEAD. To this end, the authors exploited a luciferase reporter assay in which the transcriptional activity of TEAD4 is stimulated by YAP. With this approach, verteporfin (VP), a benzoporphyrin derivative compound FDA-approved for the photodynamic therapy of ocular diseases, was identified as an efficient inhibitor of YAP/TEAD complex [85]. Further studies revealed that VP acts at different levels: in addition to inducing a conformational change in YAP and hindering its interaction with TEAD [85], VP upregulates the protein level of 14-3-3σ, which retains YAP/TAZ in the cytoplasm targeting their proteasomal degradation [132], and downregulates both YAP and TEAD mRNA levels [101]. Interestingly, VP treatment inhibited the proliferation of gastric CSCs in vitro and GC tumor growth in vivo [101]. Among the involved mechanisms, downregulation of the pro-invasive FAT1 adhesion molecule by VP has been reported [133]. However, VP is associated with non-negligible side effects and off-target activity, that reduce its therapeutic window as a specific YAP/TAZ inhibitor [134].

Super-TDU is a VGLL4 mimetic peptide that exploits the ability of VGLL4 to impair YAP-TEAD functional interaction. It was developed based on the observation that VGLL4 strongly suppresses GC growth, and tandem Tondu (TDU) domains of VGLL4 are sufficient for its inhibitory activity towards YAP. Super-TDU systemic administration markedly reduced tumor growth both in a H. pylori-infected mouse model and in GC patient-derived xenografts. Interestingly, super-TDU was more effective in GC cells with a higher YAP/VGLL4 ratio, providing clues of possible predictive biomarkers of drug sensitivity [91].

A smaller, YAP-like peptide inhibiting YAP-TEAD interaction in vitro is peptide 17 [135]. In GC cell models, this peptide reduces METTL3 expression, resulting in YAP1 downregulation and impairment of its tumor-promoting effects [104]. Unfortunately, low stability and limited membrane permeability of peptides hinder their therapeutic use in the clinical setting.

### Drugs inhibiting YAP/TAZ nuclear localization

A number of drugs have been explored relying on their ability to promote YAP phosphorylation and cytoplasmic retention. Statins are 3-hydroxy-3-methylglutaryl coenzyme A (HMG-CoA) reductase inhibitors used for lowering cholesterol levels. Studies suggested a link between statins use and reduced risk of developing GC [136, 137]. In GC cells, simvastatin impaired both Wnt/β-catenin and YAP activity, resulting in decreased proliferation and migration/invasion in vitro [138]. Mechanistically, simvastatin suppresses the activity of Rho GTPases, that results in F-actin cytoskeleton remodeling and inhibition of YAP and β-catenin signaling.

As previously discussed, STRN3 regulates the PP2A-dependent MST1/2 dephosphorylation. This concept has been exploited to develop a highly selective STRN3-derived Hippo activating peptide (SHAP). SHAP inhibits STRN3/PP2A interaction and restores MST1/2 phosphorylation, which results in YAP phosphorylation/cytoplasmic retention. SHAP administration resulted in decreased expression of YAP target genes, and reduced cell viability and tumor growth in GC models [99].

The antidiabetic drug metformin and the multi-RTK inhibitor pazopanib have also been investigated for their ability to hamper YAP/TAZ activity. In CSCs from gastric cell lines and patient-derived PDXs, metformin-induced cell cycle arrest and reduced the number of tumor-spheres, and decreased the expression of the stemness markers CD44 and Sox2. Consistently, in vivo tumor growth was delayed by metformin treatment [139]. Mechanistically, metformin induces YAP phosphorylation by AMPK-dependent phosphorylation and stabilization of the Hippo adaptor protein AMOTL1 [60]. Regarding pazopanib, this established anticancer treatment induced YAP/TAZ phosphorylation, triggering their proteasomal degradation [140]. Nevertheless, no significant signals of antitumor activity were observed in clinical trials investigating this agent in GC [141, 142].

### Drugs affecting upstream mechanical cues

In the domain of potential agents directed against mechanotransduction, attention has been focused on drugs targeting cytoskeletal tension and cell adhesion, which play a key role in regulating the Hippo pathway. FAK, a regulator of focal adhesion and cytoskeletal proteins, is involved in many oncogenic properties in GC and in other tumors [143, 144]. Recently, a mechanistic connection between RhoA and FAK was described, resulting in the activation of PI3K/AKT, β-catenin, and YAP-TAZ signaling in GC. In this context, the FAK inhibitors defactinib and PF-573228 had potent activity in GC organoids and mouse models, and reduced YAP/TAZ expression in GC cell lines [145].

Lastly, dasatinib is a small molecule used for the treatment of chronic myeloid leukemia. It targets the proto-oncogene Src and has been reported to inhibit YAP/TAZ signaling and the migration of GC cells [146, 147].

## Future perspectives

In GC, different inputs contribute to aberrant YAP/TAZ activation, and multiple connections with oncogenic pathways (e.g., Wnt/β-catenin, TGF-β) concur to the multifaceted tumor-promoting activity elicited by aberrant YAP/TAZ activity. This complexity highlights the central role played by dysregulated Hippo signaling in GC progression, distant dissemination and therapeutic resistance, making Hippo a versatile target in GC. However, the following points represent critical hurdles in the translational process: (i) The close relationship with an array of activators/inhibitors; (ii) The impact of pathway feedback loops and the mixed transcriptional outputs of YAP/TAZ (activators and repressors); (iii) The tumor-intrinsic and -extrinsic nature of the stimuli that regulate its activity, which emanate from both the local microenvironment and the systemic level.

In our opinion, the following strategies should be pursued to better frame the potential of Hippo-related biomarkers and therapeutic targeting. First, while the extensive deregulation of the pathway in GC suggests that Hippo-linked markers deserve to be investigated as prognostic/predictive factors, specific assays should be conceived by taking into account its cooperation with other oncogenic avenues (e.g. upstream regulators, mutations in components of connected pathways, and YAP/TAZ target genes). Coupling high-throughput technologies (e.g., RNA-Seq) and adequately powered studies with an identification-validation design is instrumental to provide formal evidence that Hippo signatures efficiently predict survival outcomes. At the same time, the role of mutations and copy number alterations (CNAs) hitting the pathway or its connections should be clarified to avoid overlooking the impact of genetic deregulations.

Regarding therapeutic interventions, different molecules acting at various levels of the signaling cascade have been proposed. Regardless of the specific mechanism of action, YAP/TAZ inhibitors should be developed in association with companion biomarkers. Indeed, avoiding “all-comers” studies is necessary to limit the high drug attrition rate characterizing investigational anticancer agents. An example is represented by the predictive potential of the YAP/VGLL4 ratio towards Super-TDU efficacy. Rationally designed trials aimed at detecting pathway modulation might help determine the therapeutic potential of putative YAP/TAZ inhibitors. To this end, window-of-opportunity trials represent an excellent platform for these purposes. Given that these studies envision the administration of investigational agents in the period elapsing between diagnosis and surgical resection, tumor molecular analysis can be performed prior to, and following, therapy. Thus, this specific type of trial design holds the potential to provide knowledge on the molecular mechanisms underlying YAP/TAZ targeting, the magnitude of their modulation, and the anti-tumoral effects elicited by the treatment.

A further consideration refers to Hippo signaling dysregulation in H. pylori-induced infection. Early YAP/TAZ aberrant activation suggests a critical role for Hippo in triggering tumorigenesis. Trials exploring Hippo targeting at this early stage may provide paramount information on the role of Hippo in the evolutionary trajectories of GC, from pre-cancerous lesions to gastric cancer. In conclusion, deregulated Hippo pathway and YAP/TAZ activation have increasingly been tied to the onset and progression of GC. Nevertheless, the clinical implications of aberrant YAP/TAZ activity remain to be elucidated. A coordinated workflow envisioning biomarker validation studies and biomarkers-driven interventional trials is necessary to transfer the knowledge acquired in preclinical models to the clinical setting.

## Data Availability

All data generated or analyzed during this study are included in this published article.
